# Supplementary material for: Effective Improvement of the Oxidative Stability of Acer truncatum Bunge Seed Oil, a New Woody Oil Food Resource, by Rosemary Extract
Source: Antioxidants (Basel). 2023 Apr 6;12(4):889. doi: 10.3390/antiox12040889 (PMC10135269; doi:10.3390/antiox12040889)
Supplement: Supplementary file 1 [file antioxidants-12-00889-s001.zip › Figure S1.zip!/Figure S1.pdf]

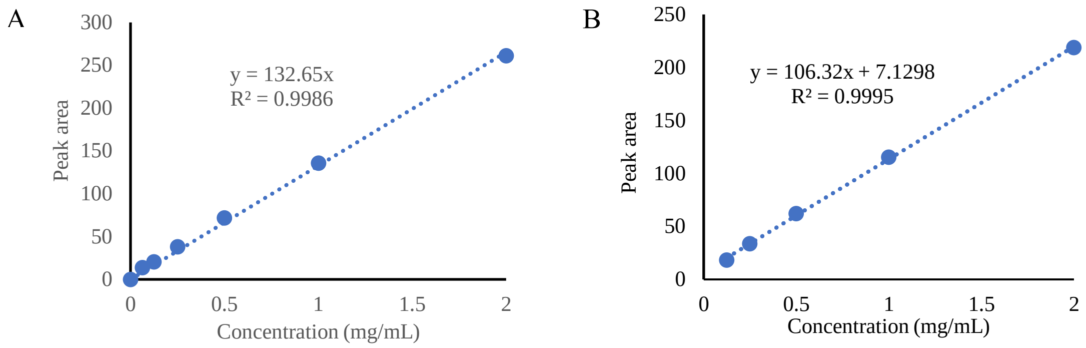

Figure S1. Standards curve for RA(A) and CA (B).
