# Supplementary material for: Effective Improvement of the Oxidative Stability of Acer truncatum Bunge Seed Oil, a New Woody Oil Food Resource, by Rosemary Extract
Source: Antioxidants (Basel). 2023 Apr 6;12(4):889. doi: 10.3390/antiox12040889 (PMC10135269; doi:10.3390/antiox12040889)
Supplement: Supplementary file 1 [file antioxidants-12-00889-s001.zip › Figure S3.zip!/Figure S3.pdf]

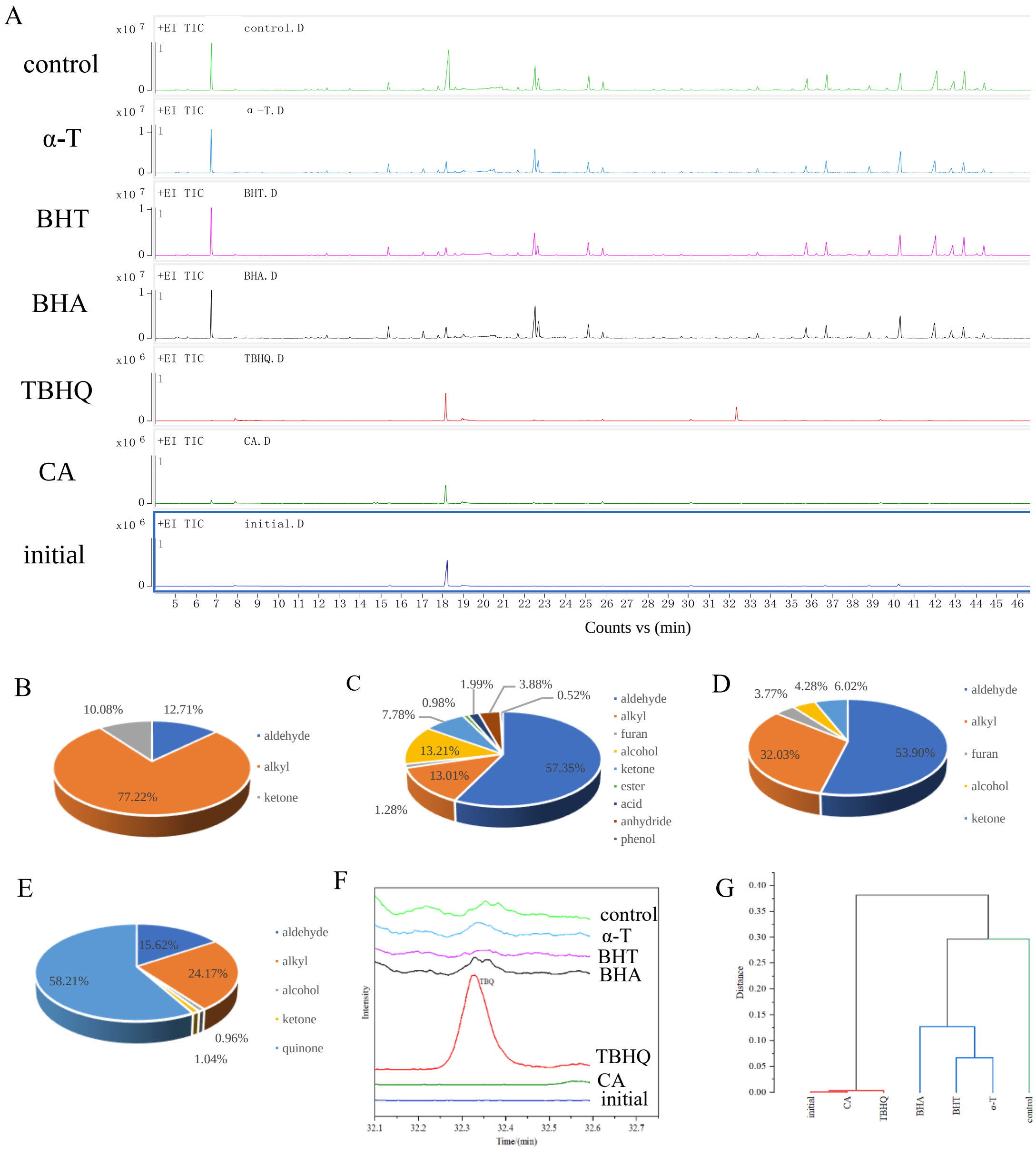

Figure S3. SPME–GC–MS spectrum of 1 g Aoil at the beginning and storage at 60 °C for 30 d (A). Volatile lipid oxidation products types of Aoil at the beginning (B) and after oxidation for 30 d (C). Volatile lipid oxidation products types of Aoil enriched with CA (D) and TBHQ (E). SPME–GC–MS spectrum of TBQ at the beginning and storage at 60 °C for 30 d (F). Dendrogram of volatiles (G).
