# Supplementary material for: Effective Improvement of the Oxidative Stability of Acer truncatum Bunge Seed Oil, a New Woody Oil Food Resource, by Rosemary Extract
Source: Antioxidants (Basel). 2023 Apr 6;12(4):889. doi: 10.3390/antiox12040889 (PMC10135269; doi:10.3390/antiox12040889)
Supplement: Supplementary file 1 [file antioxidants-12-00889-s001.zip › Figure S5.zip!/Figure S5.pdf]

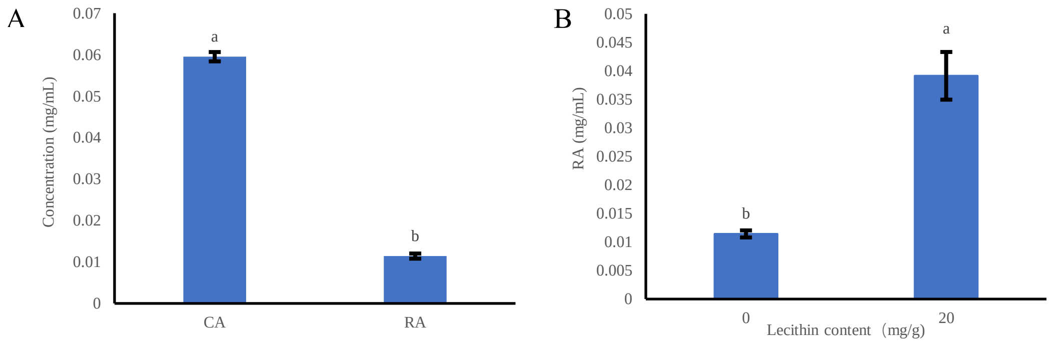

Figure S5. Determination of the solubility of CA and RA in Aoil. (A), Comparison of solubility between CA and RA in the Aoil. (B), RA concentration at different concentrations of lecithin in the Aoil.
