# Supplementary material for: Effective Improvement of the Oxidative Stability of Acer truncatum Bunge Seed Oil, a New Woody Oil Food Resource, by Rosemary Extract
Source: Antioxidants (Basel). 2023 Apr 6;12(4):889. doi: 10.3390/antiox12040889 (PMC10135269; doi:10.3390/antiox12040889)
Supplement: Supplementary file 1 [file antioxidants-12-00889-s001.zip › Table S1.zip!/Table S1.pdf]

Table S1. 37 fatty acid standards.

Number	Fatty acid
1	C4:0
2	C6:0
3	C8:0
4	C10:0
5	C11:0
6	C12:0
7	C13:0
8	C14:0
9	C14:1
10	C15:0
11	C15:1
12	C16:0
13	C16:1
14	C17:0
15	C17:1
16	C18:0
17	C18:1, trans(n9)
18	C18:1, cis(n9)
19	C18:2, trans(n6)
20	C18:2, cis(n6)
21	C18:3 n6
22	C18:3 n3
23	C20:0
24	C20:1 n9
25	C20:2
26	C21:0
27	C20:3 n6
28	C20:4 n6
29	C20:3 n3
30	C20:5 n3
31	C22:0
32	C22:1
33	C22:2

34	C23:0
35	C24:0
36	C22:6 n3
37	C24:1
