# Supplementary material for: Effective Improvement of the Oxidative Stability of Acer truncatum Bunge Seed Oil, a New Woody Oil Food Resource, by Rosemary Extract
Source: Antioxidants (Basel). 2023 Apr 6;12(4):889. doi: 10.3390/antiox12040889 (PMC10135269; doi:10.3390/antiox12040889)
Supplement: Supplementary file 1 [file antioxidants-12-00889-s001.zip › Table S2.zip!/Table S2.pdf]

Table S2. Elution procedure.

Time/min	Mobile phase A (%)	Mobile phase B (%)
0	90	10
1	90	10
8	10	90
13	10	90
13.5	90	10
16	90	10
