# Supplementary material for: Effective Improvement of the Oxidative Stability of Acer truncatum Bunge Seed Oil, a New Woody Oil Food Resource, by Rosemary Extract
Source: Antioxidants (Basel). 2023 Apr 6;12(4):889. doi: 10.3390/antiox12040889 (PMC10135269; doi:10.3390/antiox12040889)
Supplement: Supplementary file 1 [file antioxidants-12-00889-s001.zip › Table S3.zip!/Table S3.pdf]

Table S3. Fatty acid composition of Aoil.

Fatty acid composition (g/100 g)	
C4:0	-
C6:0	0.002 ± 0.0001
C8:0	0.006 ± 0.0006
C10:0	0.003 ± 0.0006
C11:0	-
C12:0	0.005 ± 0.0043
C13:0	-
C14:0	0.018 ± 0.0016
C14:1	-
C15:0	0.004 ± 0.0007
C15:1	-
C16:0	4.413 ± 0.0352
C16:1	0.092 ± 0.0015
C17:0	0.066 ± 0.001
C17:1	-
C18:0	2.536 ± 0.0141
C18:1, trans(n9)	-
C18:1, cis(n9)	11.940 ± 0.14
C18:2, trans(n6)	-
C18:2, cis(n6)	34.859 ± 0.2436
C18:3 n6	-
C18:3 n3	2.839 ± 0.0228
C20:0	0.263 ± 0.0028
C20:1 n9	8.236 ± 0.0606
C20:2	0.284 ± 0.0014
C21:0	0.006 ± 0.0007
C20:3 n6	-
C20:4 n6	0.013 ± 0.0007
C20:3 n3	0.006 ± 0.0002
C20:5 n3	-
C22:0	0.453 ± 0.0037
C22:1	17.775 ± 0.1936
C22:2	0.024 ± 0.0007
C23:0	0.030 ± 0.0012
C24:0	0.379 ± 0.0071
C22:6 n3	-
C24:1	6.327 ± 0.1549
ΣSFA	8.1837 ± 0.0666
ΣMUFA	44.3712 ± 0.4951
ΣPUFA	38.0257 ± 0.2653

Σ SFA: total saturated fatty acids, ΣMUFA: total monounsaturated fatty acids, ΣPUFA: total polyunsaturated fatty acids
